# Hypophosphates of Soda and of Lime in the Treatment of Phthisis

**Published:** 1860-07

**Authors:** Richard Quain


### HYPOPHOSPHITES OF SODA AND OF LIME IN THE TREATMENT OF PHTHISIS.

BY RICHARD QUAIN, M. D.

The treatment of phthisis by the hypophosphites of soda and of lime, was brought into notice by Dr. J. Francis Churchill, of Paris, in a communication read before the French Academy of Medicine, in July, 1857. I was at that time induced, by the representations made as to the value of these agents, to administer them to some of the cases under my care, in the Hospital for Consumption, at Brompton; but as the results were not encouraging, and as the drugs were then obtainable in limited quantities, I did not continue the experiments. One of my colleagues, Dr. Cotton, about the same time, or soon after, made some observations on the subject, and and published the results, which were unsatisfactory.

Dr. Churchill subsequently brought his memoir, with additions, before the profession. A perusal of this treatise,* and inquiries addressed to me from time to time by professional friends, as to my opinion of the value of the hypophosphites in the treatment of phthisis, have led me to re-examine fully into their asserted efficacy, and in this communication I propose to give, briefly the results of the inquiry.

It will, perhaps, be fair to say, in the first instance, that Dr. Churchill states that he was led to adopt the use of the hypophosphites in consequence of his belief that the tuberculous diathesis depended on some disturbance in the process of sanguification; that this disturbance, which affected the inorganic and not the organic elements, was due to a deficiency and not to an excess of some one or other of these elements. He argued with himself, that it could not be the sulphur, the iron, the chlorides, or the alkalies, for these substances were daily used as remedies, without any real effect on the disease. Eliminating, then, the elements first-named, he concluded that the failure was in phosphorus, as a constituent of the body.

It should here be noticed that these propositions of the author can only be regarded as theortical speculations, inasmuch as they are unsupported by either chemical or physiological observations.

By a similar course of reasoning, but one more in accordance with physiological facts, Dr. Churchill arrived at the conclusion, that phosphorus, the missing element, could be best supplied by the administration of this body in its lowest state of oxidation, as it was thereby given in a form more capable of assimilation. With that view, he adminstered the hypophosphites of soda and of lime, which he declared to be prophylactic, and to be curative in every stage of the disease. He says: "I *know* that they will prove, not only as sure a remedy in consumption as quinine is in an intermittent

---

* De la Cause Immediate et du Traitement Specifique de la Phthisic Pulmonaire. Par J. F. Churchill, D. M. P. Paris: Masson.

fever, but also as effectual a preservative as vaccination in small-pox."

Encouraged by statements like this, and by a lengthened catalogue of the phenomena of improved health which it was said resulted from the use of these remedies in Dr. Churchill's hands, I determined on giving them a fair trial in a certain number of cases. They were therefore administered in twenty-two cases, taken without selection from among the ordinary in-patients of the Brompton Hospital. Of this number (twenty-two) twelve were males and ten were females.

*The Stage of the Disease.*—Two cases were in the first, ten in the second, and ten in the third stage of phthisis.

*The Dose of the Remedy.*—Dr. Churchill recommends ten to thirty grains as the dose, of either the hypophosphite of soda or of lime, daily, in any simple fluid. The dose to be increased, until the general symptoms disappear. In some cases he prefers the one salt to the other. For example, he thinks that the salt of lime checks the expectoration, and increases the cough; whilst the salt of soda is less energetic in its action. I met with nothing confirmatory of this impression. The dose given to the patients at Brompton was, in the first instance, ten grains, three times a day, except in the case of a child, when only five were given. The disease progressing, or being stationary, or the effects of the remedy being *nil*, the dose was gradually increased. Thus, in four cases, it was increased to a drachm three times a day; in ten cases, the dose reached two scruples or more; in eight the dose remained under half a drachm. It will thus be seen that the remedy was given freely. In no case, let me add, was there any appearance of the troublesome symptoms indicated by Dr. Churchill as following large doses.

*The Duration of the Treatment.*—One case was under treatment for six months, one for four months, six for three months, nine for two months, five for one month. During this lengthened course of treatment, I looked anxiously, but in vain, for those marked physiological effects described by Dr. Churchill. There were no evidences of the "improved powers of innervation;" "the hair and nails did not grow more rapidly; there was no "appearance of plethora or of fulness;" the patients did not describe "an unaccustomed sensation of feeling better and stronger after a few doses of the remedy." Nay, I would say that there was nothing more felt by the patient nor noticeable by the physician than if so many grains of carbonate of soda or prepared chalk had been taken.

*The Results.*—To return, then, to the more immediate object for which these agents were administered, viz: to ascertain their value in the cure of consumption ; I have to state, that of the twenty-two cases, six were more or less improved while under treatment. Of these six, three were improved in but a slight degree, and only for a short time; in three the improvement was marked, but in one only of the latter has the improvement been permanent; of the two other cases, one continued using the hypophosphates for three months after leaving the hospital, during which time she grew gradually weaker, and finally died; the other, a man, after leaving the hospital, continued the treatment for some time, but gradually grew worse, and is now dying. All the other sixteen cases steadily lost ground whilst using the hypophosphites in the hospital. Happily in six of these cases, the treatment by hypophosphite was suspended, and the usual treatment by cod-liver oil, tonics, &c., being substituted, a decided improvement in each was the result.

*Conclusions.*—Reviewing the cases of which the preceding may be said to be types, we see that of twenty-two individuals laboring under phthisis, submitted to the hypophosphite treatment, sixteen derived no benefit whatever; in three the benefit was so slight and temporary as scarcely to deserve notice; in two the improvement, though marked, was temporary; and in one case the result has been satisfactory and permanent. Small as the therapeutical powers of the hypophosphites are shown to be by these facts, are we justified in assigning to them even thus much? I think not. For we cannot forget that our cases are hospital cases; that, oppressed by sickness, care, and anxiety, they come from close, unhealthy localities; that they were, more or less, destitute of good food and good air. When they enter the hospital they begin to feel the influence of hope; they live in warm, airy, and well-ventilated wards, find agreeable occupations, and have plenty of good food. Under such circumstances, the patients frequently improve in health, without the application of any medicinal agents. It would therefore be as fair to attribute the slight or temporary improvement which took place in some of these cases to hygienic as to therapeutical agencies. Nay, further; this opinion is confirmed by the fact that two of the three cases which did best in the hospital ceased to do well when they left it.

Desirous of otherwise testing the value of these substances, I thought it would be well to compare the results of my ordinary hospital practice with that of the hypophosphite treatment.

With this view, I requested my friend and late clinical assistant, Dr. Hill, (to whom I am indebted for much assistance in this inquiry, and for the notes of the preceding cases), to make abstracts of any 22 sucessive cases in the hospital books. He did so, and having ascertained the results of the treatment in these cases, I find that he has given me notes of 11 males and 11 females, of whom 3 were in the first stages of the disease, 5 were in the second, and 14 in the third. It will be remembered that 12 were in the first and second stage, and 10 in the third. Thus in the former cases, the advantage was in favor of the hypophosphite cases, so far as the stages of the disease were concerned; nevertheless we find that of the cases submitted to other treatment, 16 were more or less materially or permanently benefited, whilst in 6 only did the disease progress unfavorably. Exactly the converse was the case when the hypophosphites were given. Thus there were 16 of 22 cases unrelieved. This comparative evidence is further strengthened by bearing in mind that 6 of the cases in the former series, which were unrelieved by hypophosphite treatment, did well subsequently under other treatment.

A review of the preceding facts has led me to form a most unfavorable opinion of the value of hypophosphites in the treatment of phthisis. I believe them to be comparatively, if not absolutely useless. I have been induced to take some little pains in investigating the subject, because of the unhesitating confidence with which their value is asserted and their use recommended in certain quarters, and I have also seen in the cases of some patients who have visited Paris how much time has been thrown away by substituting the use of these salts for remedies of undoubted efficacy in controlling the progress of phthisis.—*London Lancet.*

